# Surgically Treated Central Giant Cell Granuloma in Six-Year-Old Child: A Case Report

**DOI:** 10.5005/jp-journals-10005-1208

**Published:** 2013-08-26

**Authors:** Tarasingh Patloth, J Sharada Reddy

**Affiliations:** Assistant Professor, Department of Pedodontics and Preventive Dentistry, SVS Institute of Dental Sciences, Hyderabad, Andhra Pradesh, India; Professor and Head, Department of Pedodontics and Preventive Dentistry, Government Dental College and Hospital, Hyderabad Andhra Pradesh, India

**Keywords:** Central giant cell granuloma, Maxilla, Partial maxillectomy

## Abstract

Central giant cell granuloma (CGCG) is a benign intraosseous lesion of the jaws that is found predominantly in children and young adults. Although benign, it may be locally aggressive, causing extensive bone destruction, tooth displacement and root resorption. The common therapy is aggressive curettage, peripheral ostectomy or resection, which may be associated with loss of teeth and in younger patient's loss of dental germs. In this article, a 6-year-old girl with CGCG is successfully treated surgically and this treatment is discussed along with review of the literature.

**How to cite this article:** Patloth T, Reddy SJ. Surgically Treated Central Giant Cell Granuloma in Six-Year-Old Child: A Case Report. Int J Clin Pediatr Dent 2013;6(2):146-149.

## INTRODUCTION

Central giant cell granuloma (CGCG) is defined by the World Health Organization as an intraosseous lesion consisting of cellular fibrous tissue that contains multiple foci of hemorrhage, aggregations of multinucleated giant cells and occasionally trabeculae of woven bone.^1^ Its etiology is unknown, but most of the authors consider the lesion to be an unusual proliferative response of the tissues to injury caused by trauma or inflammation.

It is an uncommon lesion that accounts for less than 7% of all benign lesions of the jaws in tooth bearing areas.^2^The mandibular/maxillary ratio has been variously reported in literature ranging from 2:1 to 3:1.^3^

CGCG commonly occurs in children and young adults with a slight predilection for females. The anterior portion of the mandible has been identified as a more common location for CGCG development with the lesion frequency crossing the midline. Radiographically, the majority of CGCGs present as an expansile radiolucency, either unilocular or multilocular, which is generally traversed by bony spicules.

Based on the clinical behavior and radiographic features, CGCG is classified as following;

1. *Aggressive lesion:* They are found in young patient characterized by rapid growth, pain, expansion and/or perforation of cortical bone, induce root resorption and high recurrence rate.^4^
2. *Nonaggressive lesion:* It is characterized by slow growth that does not perforate the cortical bone or induce root resorption and has a low recurrence rate.^5^

The traditional treatment of CGCG of the jaws has been surgical excision either by curettage or enbloc resection,^6^depending on following factors: Aggressive *vs* nonaggressive behavior, location, size and radiographic appearance. Other treatments have included radiation and systemic injections of calcitonin and interferon.

The purpose of this case report is to highlight the surgical approach used for the treatment of CGCG affecting the maxilla in a 6-year-old girl.

## CASE PRESENTATION

A 6-year-old female patient reported to the Department of Pedodontics and Preventive Dentistry, Government Dental College and Hospital, Hyderabad, with a complaint of swelling in the right side of the upper jaw. It was first noticed 6 months before the initial consultation. It was smaller in size and then suddenly increased to present size within 10 days. There was no history of fever, pain, sensory disturbance, bad taste or traumatic injury except discomfort and difficulty in mastication due to large swelling.

**Fig. 1:**
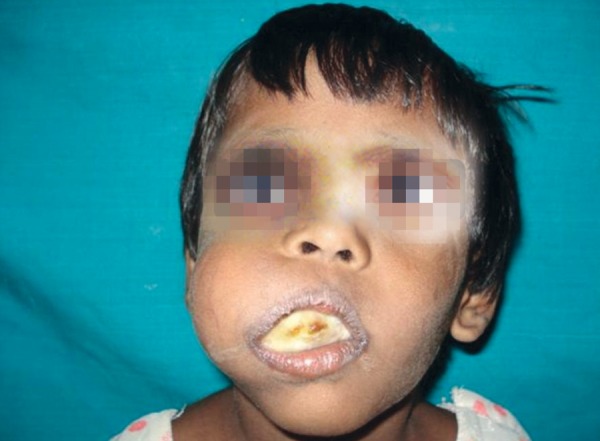
Preoperative photograph showing massive swelling involving right maxilla

Extraoral examination revealed an enormous swelling on the right side of the face involving the right maxilla causing obliteration of nasolabial fold and crossing the midline resulting in facial asymmetry (Fig. 1). The swelling had no localized elevation of temperature. There was no associated lymphadenopathy.

Intraoral examination revealed diffuse swelling on the right side of maxilla obliterating the right buccal vestibule and crossing the midline extending up to deciduous lateral incisor of contralateral side. No teeth were visible intraorally on the effected side presumably due to pathological resorption caused by the lesion leading to loss of teeth.

**Figs 2A and B:**
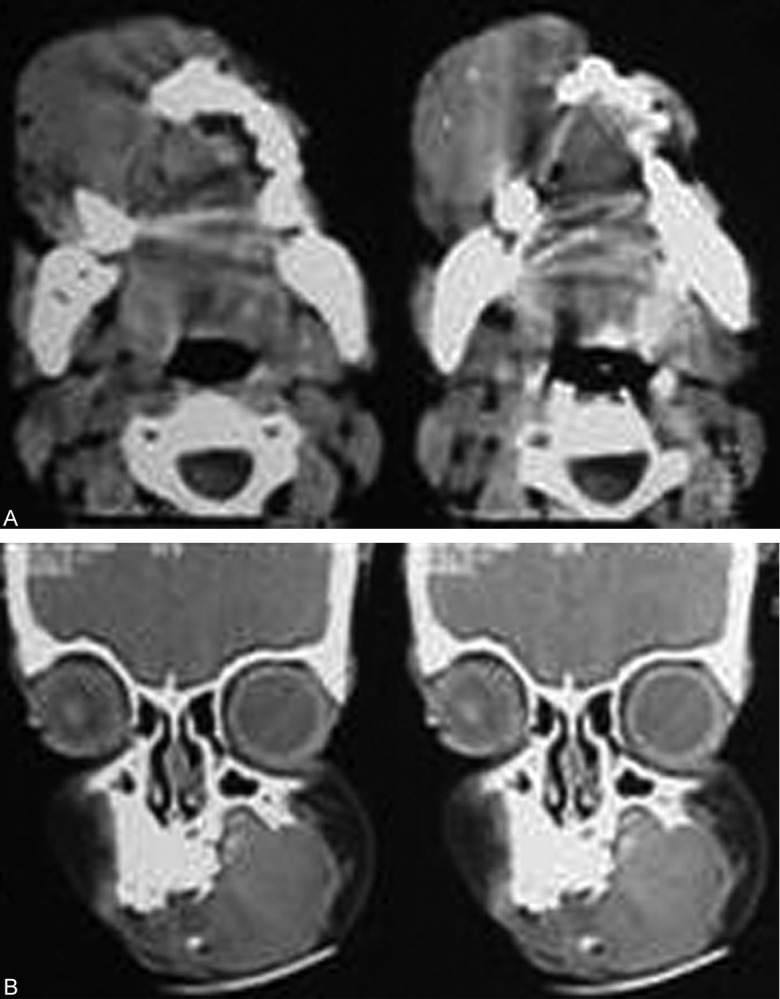
CT scans showing an expansile lesion eroding the buccal cortical plate of right maxilla

The serial axial and coronal sections of computed tomography revealed lytic lesion causing destruction of cortex in right maxilla along with overlying large soft tissue component which was benign in nature (Fig. 2A and B).

Laboratory values for serum calcium, phosphorous, alkaline phosphatase and parathyroid hormone were within normal limits.

**Fig. 3:**
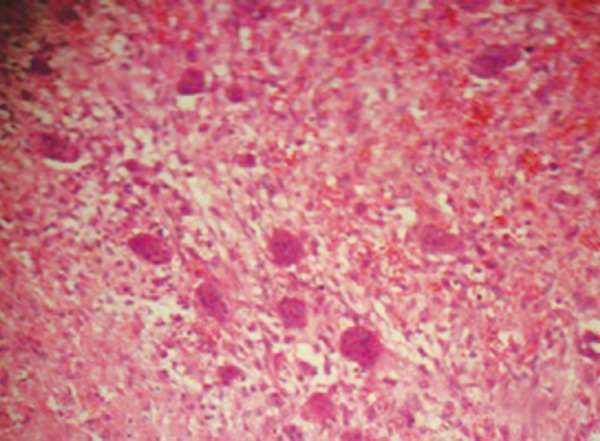
Multinucleated giant cells with dense inflammatory infiltrate

Incisional biopsy obtained showed highly cellular tumor with nodular surface folds. The ulcer base revealed granulation tissue and dense chronic inflammatory infiltrate. The cellular lesion consisted of polygonal to spindle cells arranged in sheets and bundles admixed uniformly with many osteoclastic giant cells. The giant cells showed moderate amount of fibrillary pale vacuolated cytoplasm and oval to plump elongated vesicular nuclei. Mild to moderate nuclear pleomorphism and small nucleoli with increased mitotic activity in focal areas was suggestive of CGCG (Fig. 3).

**Fig. 4:**
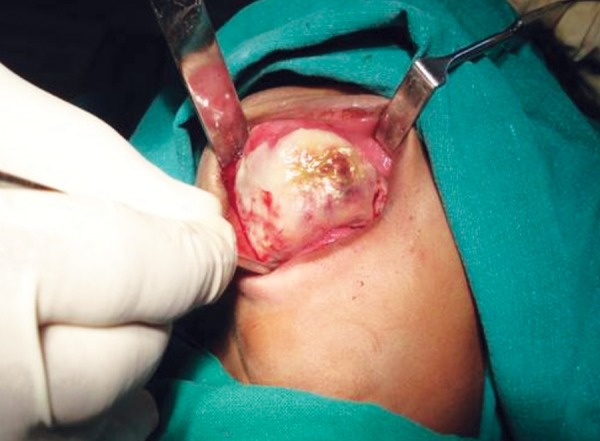
Isolated lesion intraorally under general anesthesia

Surgical approach was preferred because of the size of the lesion (Fig. 4). Partial maxillectomy was performed via an intraoral approach under general anesthesia. As all the soft tissues involved in the lesion had to be removed it became imperative that soft tissue incisions were made through them down to the bone. To facilitate this, a sharp probe was used to determine and mark out the extent of the bony defect; incisions were subsequently made at least 1 cm away from the margins of the bony defect.

**Fig. 5:**
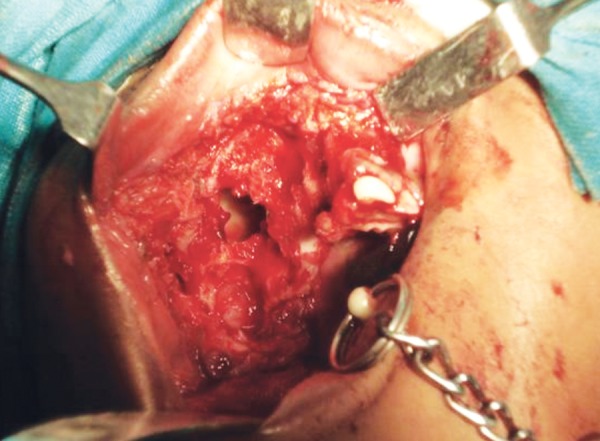
Partial maxillectomy was performed via intraoral approach

**Fig. 6:**
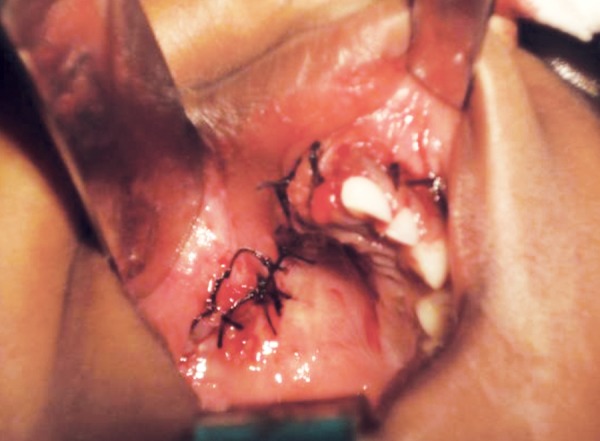
Sutures placed after the surgical excision of the lesion

**Fig. 7:**
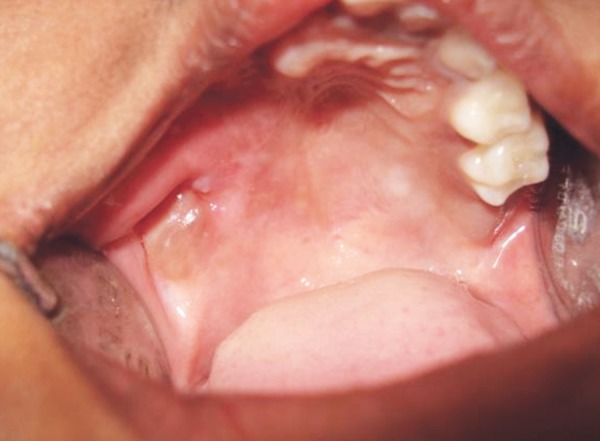
Postoperative photograph after 6 months showing uneventful healing

After reflection of the soft tissue a fissure bur was used to cut the cortical bone around the lesion approximately 0.5 cm from its margin. The lesion was reflected *in toto* with the associated tissues and was removed completely (Fig. 5). Sutures were placed and patient was recalled after 2 weeks for suture removal (Fig. 6). There after she was recalled after every 1 month. The lesion healed completely after 6 months (Fig. 7) following which reconstructive procedure was carried out uneventfully.

## DISCUSSION

The CGCG may occur at any age but it is most commonly seen in the first 3 decades. The anterior part of the mandible has been identified as the most common location for CGCGs, with some crossing the midline. CGCGs have been thought to arise in regions of the jaw originally housing deciduous teeth.^7^

CGCG of the jaw is usually unifocal. Multifocal lesions should alert the clinician to the possibility of hyperthyroidism or if bilateral cherubism. CGCG should also be distinguished from giant cells tumor of long bones. The later is locally aggressive with a high recurrence rate and a potential for malignant transformation. Some CGCGs of the jaws, despite an innocent histologic appearance, show an aggressive behavior and a tendency to recur. Ficarra et al^8^ considered that these lesions should be defined as aggressive giant cell granuloma of the jaws.

Aggressive CGCG has a tendency to recur if inadequately removed and high recurrence rates have been reported. It has been shown that recurrence usually happens when the lesion perforates the cortical plates to involve the surrounding soft tissue.^9^

CGCGs have traditionally been treated surgically. The common therapy is curettage or resection. Eisenbud et al^4^advocated the technique of curettage or curettage plus peripheral ostectomy for the treatment of CGCG. Chuong et al^5^ suggested that aggressive tumors that present with pain, rapid growth and facial swelling or cortical perforation be treated with enbloc resection.

In an attempt to distinguish aggressive and nonaggressive subtypes of CGCG and to predict the prognosis of newly diagnosed CGCGs, numerous studies have been conducted using cytometric and immuno-cytochemical methods. It has been shown that aggressive types have a higher number and relative size index of giant cells and a greater fractional surface area occupied by giant cells.

Surgical treatment of CGCGs can be associated with recurrence and serious facial mutilation and loss of teeth and tooth germs are also unavoidable. For these reasons a nonsurgical approach was suggested by Jacoway et al^10^by administration of intralesional corticosteroids. Other nonsurgical treatments include calcitonin treatment and use of alpha interferences. However, in this case nonsurgical approach was not recommended due to aggressive behavior, location, size of lesion and rapid expansile destructive growth hence surgical resection, i.e. partial maxillectomy was preformed.

## CONCLUSION

The CGCG is usually asymptomatic and is most often discovered on a routine radiographic examination and its diagnosis is confirmed by histopathological examination. The lesion can be accompanied by pain, resorption or root displacement. This aggressive lesion is usually found in young children. Our case report along with the review of literature highlights an unusual presentation that it occured in maxilla which is an uncommon site. Due to its extreme aggressiveness, among the various treatment modalities the surgical approach became essential and hence partial maxillectomy was performed.
